# CHARACTERISTICS OF LEADERS OF AGING CENTERS

**DOI:** 10.1093/geroni/igac059.1340

**Published:** 2022-12-20

**Authors:** Neil Charness

**Affiliations:** Florida State University, Tallahassee, Florida, United States

## Abstract

In December of 2021 and January of 2022, current leaders of Centers on Aging belonging to the GSA Age Directors interest group were invited to fill out a survey inquiring about their backgrounds, training for leadership, and perceived needs for training via an online Qualtrics survey. Thirty-one responses were received though a few responses were missing for most questions. The sample mostly resided in the USA (86%), and identified mainly as female (63% female, 37% male). Most were married (90%). The average age of a respondent was 58 years (SD=9; range 37-74). Respondents were not diverse: 97% White. Most had doctoral level education (1 Master’s level). The results indicate that there is a need to enhance and develop leadership skills in diverse mid-career gerontologists in order to provide replacements for an aging cohort of directors. Results are also consistent with prior National Academies’ recommendations for training the gerontological workforce.

